# Pharmacist Prescribing for Minor Ailments Service Development: The Experience in Ontario

**DOI:** 10.3390/pharmacy9020096

**Published:** 2021-04-27

**Authors:** Nardine Nakhla, Anastasia Shiamptanis

**Affiliations:** 1 School of Pharmacy, University of Waterloo, 10A Victoria St. S., Kitchener, ON N2G 1C5, Canada; 2 Ontario College of Pharmacists, 483 Huron St., Toronto, ON M5R 2R4, Canada; anastasiapharmd@hotmail.com

**Keywords:** minor ailments, minor illness, self-limiting conditions, low acuity conditions, self-care, ambulatory conditions, pharmacist prescribing, scope of practice, advanced pharmacy practice, access to care

## Abstract

To date, eight of ten Canadian provinces have authorized pharmacists to prescribe for minor ailments. Prompted by a request by the Ontario Minister of Health, draft regulations were submitted to enable this pharmacy service in Ontario. Differences exist in how jurisdictions have approached development and delivery of these programs. This paper will summarize key differences and similarities among existing programs while highlighting the multi-pronged approach utilized by Ontario. Such an approach involved broad stakeholder engagement, implementation science, and an evaluations framework to guide an assessment of the impact of this new service. These insights can be leveraged by other jurisdictions planning to initiate or evolve their minor ailment prescribing services.

## 1. Introduction

The current COVID-19 pandemic has highlighted the unprecedented need to consider more multifaceted, sustainable solutions for healthcare globally. Pharmacists are Canada’s third largest group of healthcare professionals (HCPs), with approximately 45,651 licensed pharmacists across the nation [1]. Pharmacists possess extensive knowledge and skills in pharmacotherapeutics that can be optimized to narrow disparities and facilitate access to high-quality healthcare. For example, pharmacists have a long history of helping patients manage minor ailments. Traditionally, this has involved making non-prescription medication recommendations, facilitating other self-care decisions (e.g., lifestyle changes), and referring patients to other HCPs when needed. As one of the most accessible HCPs, pharmacists are well-positioned to strengthen community-based care for a variety of conditions. Canadian estimates show that 10 to 30% of physician consultations are for minor ailments [2]; United Kingdom (UK) estimates are higher, suggesting that between 18% and 37% of general practitioner consultations are for minor ailments, many of which can be managed by pharmacists [3]. 

In recent years, several countries introduced “pharmacist prescribing for minor ailments” (PPMA) in an effort to increase access to primary healthcare and reduce the burden of minor ailment care on other settings (e.g., emergency departments (EDs), urgent care) by leveraging the clinical capabilities of pharmacists and providing patients the choice of receiving care from community pharmacists [4,5]. PPMA programs promote efficiencies within the healthcare system by enabling pharmacists to prescribe or recommend the most appropriate treatment for certain minor ailments, reducing the need to refer to another HCP and setting for a prescription [6]. This affords patients with the opportunity to receive timely care in the community from highly accessible and trained HCPs. A systematic review by Paudyal et al. concluded that the low reconsultation and high symptom-resolution rates of up to 94% associated with PPMA programs suggest that these services are being appropriately managed by pharmacists and do not compromise patient safety or quality of care [4]. Furthermore, a 2014 review supported community pharmacy-based minor ailment services as an effective and efficient strategy for managing patients that may reduce strain on healthcare systems and thus facilitate sustainability [7,8].

PPMA programs exist in some Canadian provinces and in some parts of the UK (i.e., Wales, England, Scotland, and Northern Ireland) [2,9,10,11,12,13,14,15]. Their feasibility is currently being considered in Spain, New Zealand, and Australia [16]. The term “pharmacist prescribing for minor ailments” (PPMA) is commonly used across Canada to depict the community pharmacy service that entails assessment and prescribing of medications to manage minor ailments. However, the term “minor ailment service” (MAS) and “common ailment schemes” (CAS) are used to characterize similar services globally. For this paper, the term PPMA is used interchangeably for all regions and countries.

PPMA services differ in their structural design and service delivery regulations from country to country and even from jurisdictions within the same country [2,6]. Aly et al. recently performed the first international comparative review of minor ailment service features and outlined essential elements to consider when designing such a program [6]. At the time of publication, Ontario submitted draft regulations to the government to enable this type of pharmacy service. The aim of this paper is to describe the process utilized by Ontario, which incorporates data analysis, broad stakeholder engagement, patient involvement, implementation science, and an evaluations framework. Through this experience, we build on the work done by Aly et al. and propose additional factors to consider when developing and implementing PPMA programs [6].

## 2. Ontario: Constructing the Approach

In May 2019, as part of an overall approach to expand the scope of HCPs, the Minister of Health directed the Ontario College of Pharmacists (the College) to submit draft regulations by June 2020 to enable pharmacists to prescribe drugs for certain minor ailments to improve access to routine care for patients in the community and reduce the need for emergency or urgent care visits [17]. This request occurred after a 2019 report commissioned by the government, Improving Healthcare and Ending Hallway Medicine, that provided recommendations on creating a more efficient healthcare system in which patients can access care where and when it is needed [18]. Soon thereafter, and consistent with the College’s mission of driving quality and safe pharmacy care and improved outcomes, the College developed a multi-pronged system- and outcomes-focused approach to drafting the regulations. This included working closely with the Ministry of Health throughout the process to align policy direction and key regulatory changes. In positioning the PPMA program to meet the Ministry’s objective of providing more pathways for patients to access care in the community, the College felt it was important to consider several factors, including experiences from other jurisdictions, the perspectives of those most impacted by these changes (i.e., patients, pharmacists, physicians), and the healthcare system as a whole.

The key pillars of the multi-pronged approach will be described below and included defining the regulatory framework, establishing an expert group, and patient involvement and broad engagement.

### 2.1. Defining the Regulatory Framework

In Ontario, regulations to enable pharmacists prescribing for smoking cessation were added to the *Pharmacy Act, 1991* in 2012. As such, a framework on general expectations for prescribing was devised at that time. This included notifying the primary care provider (PCP) after prescribing, providing the patient with the option of dispensing the medication at another pharmacy, and assessing documentation expectations. The amendments are therefore built on the existing regulations related to prescribing for smoking cessation. The provisions were reviewed to determine if additional prescribing expectations specific to minor ailments were needed. Additionally, it was important to consider if there are any existing regulatory provisions that may impact the policy direction. For example, in Ontario, the College has the authority to enable pharmacists to prescribe specific medications or medication categories, whereas other provinces allow for broader prescribing.

In a jurisdiction lacking the foundational framework for policy development, we suggest drawing from the profession’s standards of practice, code of ethics, and any applicable practice directives (e.g., criteria for prescribing, dispensing) issued by the regulatory authority to develop the basis of such a service. Stakeholder engagement is also key in identifying any safeguards needed and refining practice expectations if necessary.

### 2.2. Establishing an Expert Advisory Group

To facilitate expedient and systems-focused discussions, the College assembled a Minor Ailments Advisory Group (MAAG) composed of patient advocates, community pharmacists, physicians, academics, and health systems research experts soon after the Minister’s request was made. The expert advisory group provided advice and input to the College on key regulatory considerations and safety parameters to align the program with the government’s objectives while maintaining quality patient care. This involved working on various aspects of planning and development, including draft regulations, proposed minor ailments and associated medication categories, safeguards for implementation, an evaluations framework for prescribing, and practice support tools [19].

The multi-stakeholder composition of MAAG facilitated robust discussions. The group held regular meetings over the course of several months (June 2019 to November 2020) and followed a structured, modified-Delphi approach in providing their advice and guidance to the College on which minor ailments and medication categories to include. Key inputs in the decision-making process included the results of an environmental scan, experience in other provinces, ED administrative data on conditions that potentially could be managed by a pharmacist, and feedback from pharmacy professionals and patients obtained through various mechanisms such as surveys and focus groups. Criteria were agreed upon by MAAG to apply a consistent approach in evaluating which minor ailments to recommend for the minor ailment program [19]. The key actions taken by MAAG are summarized below.

#### Environmental Scan and Assessment

MAAG contributed to an environmental scan, which included review of quantitative and qualitative data to explore the current landscape of pharmacist prescribing for minor ailments across various Canadian jurisdictions. Additional data were also collected from the websites of the provincial regulatory authorities as well as provincial and national pharmacy advocacy groups. Through this review, the group considered all of the minor ailments included in any provincial program and studies relating to evaluation of these services. It was noted that terminology of the service and criteria for inclusion varies across jurisdictions, and that there was no universally accepted definition for minor ailments. Table 1 provides a breakdown of differences in prescriptive authority and terminology used across Canada.

It was the consensus of the MAAG to align the term “minor ailment” with the definition used in Nova Scotia and Newfoundland and Labrador, as well as those found in the literature [16,20,21,22]:

Minor ailments are health conditions that can be managed with minimal treatment and/or self-care strategies. Additional criteria include:

- usually a short-term condition;
- lab tests are not usually required;
- low risk of treatment masking underlying conditions;
- medication and medical histories can reliably differentiate more serious conditions;
- only minimal or short-term follow-up is required.

Definitions which included the term “self-limited” (e.g., Saskatchewan, Manitoba) were not adopted in their entirety as some conditions, such as urinary tract infections, may not fully resolve without treatment. Any condition that did not meet this definition were not considered by MAAG for inclusion under these regulations.

The studies identified from the environmental scan were used to draw upon the experience of other Canadian provinces in implementing PPMA programs. MAAG applied learnings from the experiences in other jurisdictions in making recommendation for the program. For example, the main reported barriers to adoption of PPMA services were integration into workflow, patient awareness, lack of sufficient revenue, and lack of time [23,24]. In alignment with the Institute of Healthcare Improvement Quadruple Aim, MAAG reviewed studies reporting on the impact on patient experience and health system efficiency [25,26]. Patients avoided physician and ED visits as the result of provincial PPMA programs [27] and were able to access care sooner [28]. Patients reported that they are satisfied with the service [27,29,30]. 

Key findings from the environmental scan were applied to the Capabilities, Opportunities, Motivation, and Behavior model (COM-B) to assist MAAG with identifying and recommending strategies for change, including regulation, policy, implementation, and evaluation. Refer to Section 3.1.3 for additional information. 

Additionally, information on conditions that are resulting in ED visits that can be safely managed through pharmacist prescribing was also reviewed to help ground the policy in current and emerging healthcare needs and maximize the benefits to patients and the broader health system [31].

**Figure A1:**
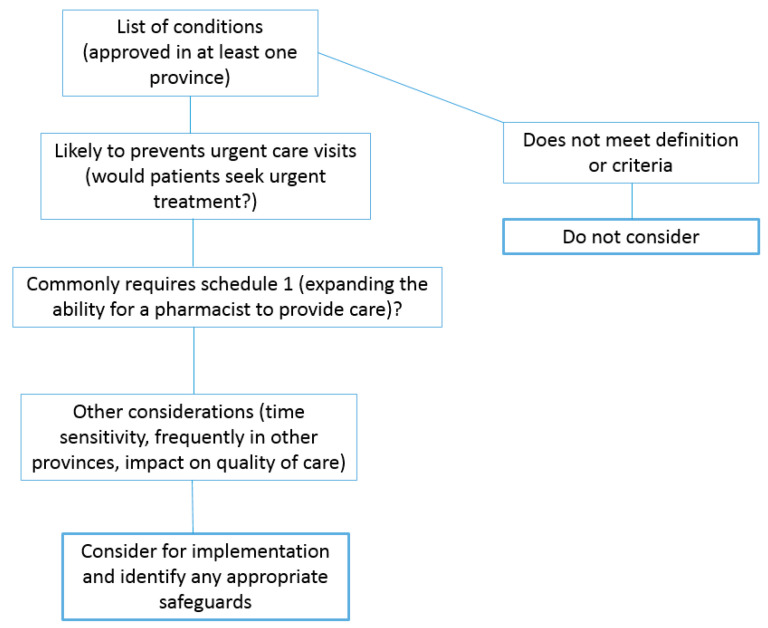
Conceptual framework.

After building this evidence-based foundation, MAAG used a conceptual framework (Appendix A Figure A1) with criteria to guide decision-making for selecting which minor ailments to recommend for inclusion in the PPMA program. The criteria included: likelihood of preventing urgent care visits; treatment commonly involves the option of a prescription medication; time-sensitive conditions; and those frequently included in other provinces. The MAAG followed a three-round modified-Delphi approach in reviewing the minor ailments. First, each MAAG member utilized the criteria and prioritized the conditions based on high, moderate, and low priority for implementation, and those that do not meet the definition. The collated results were shared with the group, and a second round of prioritization occurred via an anonymous web-based questionnaire. After this second round, conditions with variation were discussed by the group and final agreement was then obtained at an in-person consensus meeting (round three). Through this process, MAAG refined the full list of conditions approved in at least one province to a list of 18, which were circulated for broad feedback. 

A similar three-round modified-Delphi approach was taken to provide recommendations for medication categories. The MAAG reviewed the literature and guidelines to identify the most common treatment options for the minor ailments and considered both the risk level of the medications and the level of follow-up required in making a final recommendation to the College. 

### 2.3. Patient Involvement

A cornerstone to the approach was the involvement of patients throughout the process. In addition to patient involvement on the MAAG, the College conducted third-party facilitated focus groups to obtain feedback on the list of minor ailments and other safety considerations. Through these focus groups, key implementation considerations were highlighted such as the importance of communication between HCPs to support continuity of care and the need for the practice environment to be conducive to the provision of the PPMA service. Additional public input was sought through the Citizen Advisory Group (CAG), a collaborative partnership that brings together patients and caregivers to provide feedback related to health regulation in Ontario [32]. The CAG provided insight on which minor ailments they would be most likely to see a pharmacist for, which included urinary tract infections, hemorrhoids, conjunctivitis, and insect bites [32]. Meaningful patient involvement was key to the regulatory development process as it directed the program to areas most important to patients.

### 2.4. Broad Stakeholder Engagement

The request to expand the scope of practice of pharmacists recognizes the role of the profession in collaborating to improve Ontario’s health system outcomes. It is therefore important to understand, from the perspective of both the public and other professions, where prescriptive authority for minor ailments for pharmacists will have the greatest impact to improve system and patient outcomes. To achieve this understanding, the College engaged extensively throughout the regulatory development process leading up to, and including, the final open consultation on the proposed amendments to provincial regulations.

The objectives of the PPMA service were shared at each point to enable stakeholders to contribute insights to help achieve the overall system goals. A survey was administered to pharmacy professionals early in the process to inform the policy direction. Pharmacy professionals and students were asked through the survey to provide feedback on the preliminary list of 18 conditions. Furthermore, the College engaged in discussions with health professional associations, including pharmacy, medicine, and nursing, and collaborated with other health regulatory colleges.

The College also obtained feedback from stakeholders through the open consultation process, which ensures that the public, practitioners, and all other interested stakeholders have an opportunity to provide feedback on new and devised documents related to pharmacy practice prior to final approval. Feedback was received from pharmacy professionals, pharmacy students, the public, associations representing pharmacists, physicians, and family health teams, as well as pharmacy organizations. Together, these perspectives provided important insights that helped to inform regulatory drafting and the implementation strategy. Further refinement to the list of medication categories also occurred based on feedback obtained [33].

## 3. Ontario: The Final Results

### 3.1. PPMA Service Components

Triangulation of the results of the environmental scan along with health administrative data and broad engagement coalesced to produce results that informed the structural design. A description of the structural design features and delivery of the PPMA service can be found below.

#### 3.1.1. List of Minor Ailments and Medication Categories

Based on the feedback received, and to align with the Ministry’s direction, the preliminary list of 18 ailments was further refined to 12:

- Urinary tract infection (uncomplicated)
- Dermatitis (atopic/eczema, allergic and contact skin rashes)
- Insect bites (including tick bites) and urticaria (hives)
- Conjunctivitis (bacterial, allergic, viral)
- Allergic rhinitis (nasal symptoms from allergies)
- Candidal stomatitis (oral thrush)
- Herpes labialis (cold sores)
- Hemorrhoids
- Gastroesophageal reflux disease (GERD)
- Dysmenorrhea (menstrual cramps)
- Musculoskeletal sprains and strains
- Impetigo (bacterial skin infection common in children)

Some of the conditions initially proposed were not eliminated entirely but rather grouped together based on therapeutic similarities. For example, prescribing for post-exposure prophylaxis of Lyme disease from tick bites was moved to the insect bites category. Tick bites was added to the list due to the importance of timely access to prophylaxis treatment and the high-risk areas for Lyme disease in the province, which include remote locations [34].

The agents authorized for pharmacist prescribing to manage the 12 ailments, if treatment is considered necessary and appropriate, are outlined in the draft regulations according to the American Hospital Formulary Service (AHFS) classification system. Considerations such as recent evidence, clinical practice guidelines, best practices, and antimicrobial stewardship guided the final selection of the medication categories. 

#### 3.1.2. Safeguards

Elements to support safe implementation identified through the consultations included collaboration, patient assessment, and consideration of any conflict-of-interest involvement in prescribing. The safeguards in place (i.e., current regulations for prescribing, standards of practice, code of ethics) in Ontario address these factors. 

Two additional elements were added to the regulation to address common feedback received around ensuring that pharmacists are aware of the practice expectations, such as referral to another HCP when appropriate. To assist pharmacists with understanding, interpreting, and applying the regulations to their practice, the College Board approved a mandatory orientation module. This short online module reinforces important practice expectations, such as referring to an appropriate HCP for patients presenting with “red flags”, and highlights the expected purposes of the service. PPMA will become a general practice expectation of pharmacists in Ontario. As such, all practicing pharmacists are required to complete this module within one year of its availability. Furthermore, previous work in this area noted that to assist with sustainability of a PPMA program, it is important for the profession to be clear on and understand the objectives of the service [35,36]. Clinical education is not mandated and, as with any practice development, pharmacists are responsible for self-assessing their need for additional continuing education to ensure that they have the required knowledge, skill, and judgement required to provide quality patient care. Both pharmacy university programs in Ontario—and all ten across Canada—include minor ailments in their curricula [37]. Additionally, Ontario pharmacies are required to have the references and resources needed for pharmacists to apply best practices and evidence-based care. Information on the assessment and treatment for minor ailments as well as algorithms are available in Canadian drug references. The orientation module directs pharmacists to these resources and reinforces expectations for the use of evidence and guidelines. The use of guidelines and algorithms to support clinical decision making were identified as important facilitators for supporting quality patient care [35]. 

Moreover, to support pharmacists in their role as antimicrobial stewards, the MAAG recommended that resources (i.e., concise practice tools) be developed to facilitate assessment and appropriate antibiotic prescribing for uncomplicated UTIs and Lyme disease post-exposure prophylaxis from tick bites in Ontario. These resources are currently under development by a MAAG subgroup. 

#### 3.1.3. Implementation and Evaluation

Key findings from the environmental scan were applied to an implementation framework (the behavioral change wheel) to assist MAAG with identifying and recommending implementation strategies.

Following a framework provides a theory-informed structure to optimize the uptake of interventions. This is done by tailoring implementation strategies to the barriers and facilitators to the behavior change. The COM-B model is central to the behavior change wheel framework. COM-B helped to elucidate the barriers and facilitators according to three determinants of behavior change, capability, opportunity, and motivation, and the subsequent identification and alignment of levers for adoption for PPMA services. Using a model enabled a more in-depth understanding of what may influence the behavior change, in this case, engaging in prescribing for minor ailments, in order to determine implementation strategies that will address those influencers or barriers and facilitators. For example, concerns related to work-load pressures and the ability to meet practice expectations are potential barriers than can relate to the opportunity domain of COM-B. According to the behavior change wheel, effective strategies should involve communication and address environmental concerns. The implementation strategy for PPMA therefore includes a robust communications plan to inform the profession and public of this service, as well as a plan to address concerns with the practice environment that were previously identified through other stakeholder engagement activities. The College initiated a community practice initiative to explore and better understand the workplace challenges and further strengthen the quality and safety of pharmacy care in the province.

**Figure 1:**
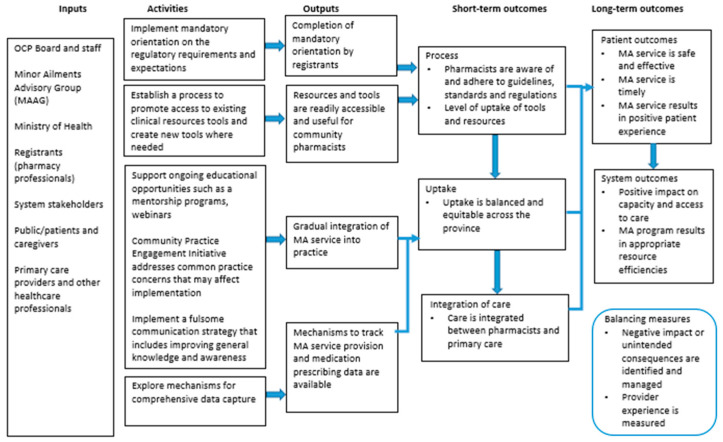
Logic Model for Pharmacist Prescribing for Minor Ailments (PPMA).

Along with an implementation plan, an evaluations framework was also developed (see Figure 1) by MAAG. The framework, depicted in a Logic Model, is intended to guide the development of a plan to measure the impact of the PPMA program on system and patient outcomes, which may involve studying symptom resolution and reconsultation rates. The results from these evaluations will help elucidate the impact on patient safety, which is critical to the determination of further scope enhancements. Additionally, consistent with continuous quality improvement principles, the evaluation may help identify opportunities to optimize how the program is administered.

## 4. Discussion

Enabling pharmacists to prescribe for minor ailments will allow them to take on a greater role within the healthcare system, to provide patients with streamlined care pathways, and to improve access to minor ailment care in the community.

While the potential benefits of PPMA services are clearly documented in the literature, there are much less surrounding optimal structures and strategies for building an evidence-based model that enables the reported positive service outcomes. PPMA services differ in their service delivery regulations and structural design from country to country and even from jurisdictions within the same country [2,6]. In this section, we will review similarities and differences between our Ontario model and PPMA services that exist in other jurisdictions.

In the UK, PPMA services authorize pharmacists to supply medications through locally agreed protocols or by supplementary and independent prescribing [3]. In Canada, Saskatchewan pharmacists must prescribe according to protocols that are established for each minor ailment. In Manitoba, pharmacists can prescribe medications in specific medication categories for specific minor ailments without a requirement to follow a standard prescribing protocol. Other provinces (e.g., New Brunswick, Prince Edward Island, Nova Scotia, Newfoundland and Labrador) allow broad prescriptive authority for the minor ailments whereby pharmacists can prescribe any medication that is indicated for the minor ailment. Alberta follows a different model and does not follow a formal PPMA program. Pharmacists may obtain an additional prescribing authority (APA) designation that permits the initiation of any Schedule 1 therapy (with the exception of narcotics and other controlled substances) for any condition. As such, Alberta does not confine pharmacist prescribing to medication categories, medical conditions, or protocols. Furthermore, there are some variations in nomenclature and classification of minor ailments. For example, Manitoba uses the term “self-limiting conditions” while Saskatchewan uses “minor ailments and patient self-care” to describe PPMA services. Differences also exist in the nomenclature of the minor ailments and in classifying which conditions are included under the PPMA service umbrella. For example, canker sores are referred to as “oral ulcers” in Prince Edward Island, “recurrent oral aphthae” in Manitoba, and “aphthous ulcers” in Newfoundland and Labrador. In some provinces (e.g., New Brunswick, Saskatchewan), urinary tract infection is included under the PPMA service, whereas in Nova Scotia, pharmacists can prescribe for the condition; however, this is outside of the minor ailment specific regulation. Currently, only Alberta, Saskatchewan, and Quebec publicly fund the service. There is therefore considerable variation nationally in how the PPMA services are designed, administered, regulated, and funded [2]. 

Similar to Aly et al., engagement with a variety of key stakeholder groups was an integral component weaved throughout the service design process [6]. This broad stakeholder engagement offered insight related to various perspectives of the key stakeholders, illuminated the potential impacts on different areas of the healthcare system, and ultimately informed the policy direction. As was found in the study exploring the perceptions of stakeholders on a national common ailment service in Wales, engaging stakeholders in the early stages of the pharmacy-based program increased sense of ownership among this group and garnered wide-spread support for the service [38]. Early engagement provided an opportunity to incorporate feedback and concerns early in the development process. For example, concerns were raised around pharmacists having the necessary education to assess and prescribe medicines for minor ailments. It was confirmed that patient assessment for minor ailments is an integral component of the core curricula at all ten faculties of pharmacy across Canada [37]; graduates are equipped with the necessary competencies to support clinical decision making required for minor ailment prescribing. In addition to curricula, continuing education for minor ailment prescribing has been available and continues to be accessed by the profession in preparation for this scope, which has been long-awaited. The mandatory orientation module reinforces the importance of referring patients to other members of the healthcare team for management of issues beyond their competence as well as the need to self-assess continually one’s knowledge, skill, and judgement necessary for the provision of safe and quality patient care.

Stakeholder feedback activities were also embedded within the structured process used to prioritize the ailments and informed refinement of the final list. This process also included analysis of data from multiple inputs, including the environmental scan, MAAG’s modified-Delphi proceedings, and provincial data describing the most prevalent minor ailments resulting in ED visits across Ontario [31]. The latter provided insight on the impact that pharmacist prescribing may have if this type of routine or minor care were moved into the community. A UK study published by Nazar et al. utilized expert consensus through interacting group decision and nominal group process [39]. The group reviewed anonymized patient data and reached consensus on low acuity conditions that could be safely redirected to the community pharmacy through the National Health Services medical helpline. This approach was similar to Ontario’s in that an expert group provided advice via consensus by analyzing data and identifying conditions that would be appropriate for pharmacist intervention. In that study, the participants were divided into smaller groups to facilitate parallel discussions to achieve consensus across groups on conditions that required additional discussion, whereas in Ontario, these discussions occurred in the larger group setting. While both considered risk to patient in the prioritization of the conditions, Nazar et al. used the failure mode and effects analysis (FMEA) method to help identify risk, whereas we relied on expert guidance and the conceptual framework to help inform these decisions. There may be value in utilizing a standard risk tool when selecting minor ailments for inclusion in PPMA programs. Further research exploring the impact of such a tool on patient outcomes would be beneficial.

The next step entailed selecting medication categories for pharmacist prescribing in Ontario. Our approach in utilizing the AHFS classification system differs from all other jurisdictions in Canada partly due to our regulatory framework, which does not allow for broad prescriptive authority as is the case in other provinces. The regulations must include either medication names or categories; medication categories were preferred over medication names as categories and offer a more flexible approach than medication lists to ensure pharmacists have access to the most up-to-date medications without requiring a regulatory change. The AHFS system was used to align with how other healthcare professions reference medications in the regulations.

Embedded within our provincial regulations, and consistent with that of most Canadian provinces, is the requirement for pharmacists to notify the PCP of the patient consultation and treatment plan after prescribing; this safeguard differs from other PPMA services globally as it is not always a mandatory element in other countries [6]. However, communication with the PCP was identified by patients during our stakeholder engagement activities as an essential component of the service for continuity of care purposes, cementing its importance in service delivery. Additionally, pharmacies in Ontario are not connected to a provincial electronic medical record, therefore setting out the added expectation for communication with the PCP. The communication channel is not specified, and pharmacists may choose a method established with the PCP.

In Canada, the training requirements to prescribe for minor ailments vary provincially; some jurisdictions (e.g., Nova Scotia) do not require any training, while others mandate a non-clinical orientation to the regulations (e.g., Ontario, New Brunswick), and others have more comprehensive, clinical training requirements (e.g., Saskatchewan) [40]. In Ontario, pharmacists will be required to complete an online orientation to the regulatory requirements and practice expectations. A recent international review examining the evidence regarding training, education, and assessment requirements associated with the delivery of PPMA services by community pharmacists and other pharmacy staff found that 46% required pharmacists to complete mandatory training, which varied in its nature, time commitment, and delivery (e.g., live, online) [35].

Globally, antimicrobial resistance continues to be a significant public health threat as antibiotics are frequently prescribed for viral illnesses where there is no benefit to patients [41]. Specifically, antibiotic overuse is associated with rising rates of drug-resistant infections, avoidable adverse drug reactions, and increasing healthcare costs [42]. U.S. data estimate that approximately 30 to 50% of antibiotics prescribed in outpatient settings may be unnecessary [43,44]. Enabling pharmacists to prescribe antibiotics to treat common infectious diseases offers an opportunity to harness pharmacists as a source of improved rational antibiotic prescribing and patient education, enhancing access to care and ensuring the right patients get antibiotics when necessary. To support pharmacists in this role, the MAAG co-developed practice tools that are based on evidence and antimicrobial stewardship principles to guide assessment and decision-making for patients with UTIs and tick bites.

The evidence of the benefits of pharmacist prescribing of antimicrobials is growing. The RxOUTMAP study found that pharmacist management of uncomplicated UTIs across 39 community pharmacies in New Brunswick was effective and safe, and resulted in high patient satisfaction [29]. Of the 750 patients enrolled, clinical cure was achieved in 88.6% of those managed by a pharmacist compared to 91.1% for those managed by a physician (*p* > 0.99), and there were no statistically significant differences in safety outcomes [29]. The time from the patient’s decision to seek care until they were seen by a pharmacist or physician was 1.7 vs 2.8 days, respectively (*p* = 0.0153). These results are consistent with previous studies on this topic. A smaller Scottish study comparing the care pathways of patients with UTI symptoms found that patients seeking pharmacist management presented earlier than those seeking physician management (*p* = 0.026), and that providing antibiotics for UTIs through strict protocols in community pharmacies improved access to treatment for patients and had the potential to maintain antibiotic stewardship while reducing physician workload [45]. Recognizing this role for pharmacists and considering the healthcare needs, Ontario included UTI in the proposed PPMA program. Other provinces (e.g., Prince Edward Island, Quebec) also recently enabled pharmacists to prescribe for UTIs. These types of evaluative studies helped to inform policy direction in terms of including conditions that involve antimicrobial prescribing.

Implementation science assists with analyzing the most important domains specific to a policy direction to determine how to execute the desired outcome successfully. In our experience, implementation science informed our approach to operationalizing the service by focusing on the key drivers of behavioral change, which includes capability, opportunity, and motivation [46]. During the early engagement activities, stakeholders were surveyed to provide feedback on potential facilitators and barriers to incorporating PPMA into their practice. Coupled with the findings from the environmental scan, the resultant factors were then mapped to the COM-B framework to enhance understanding of the complexity of potential facilitators and barriers, and ultimately identify strategies to assist with implementation. For example, integrating PPMA into practice in a busy environment was noted as a concern by members of the public and pharmacy professionals, which is consistent with the literature [23,47]. Applying this identified barrier to the framework allowed for recognition of the importance of creating an opportunity for these services by making the practice environment more conducive to this type of service.

The COM-B model can be further elaborated by the Theoretical Domains Framework [48], which consists of 14 domains covering the spectrum of behavioral determinants. These domains can be mapped directly onto the COM-B components. In their study, Isenor et al. utilized the Theoretic Domains Framework version 2 to identify the relationship between barriers and facilitators to pharmacist prescribing and self-reported prescribing activity [49]. The three domains that respondents most positively associated with prescribing were knowledge, reinforcement, and intentions. The largest effect on prescribing activity was the skills domain. The study concluded that this understanding could assist the development of policy and program interventions at the pharmacist, pharmacy, and health system levels, to increase the uptake of pharmacist prescribing.

Currently, there seems to be limited evidence about the effectiveness of tailored implementation strategies. In Ontario, the evaluation framework was constructed alongside the implementation strategy in order to evaluate both the outcomes of the PPMA program and the process. The framework will be used to guide evaluators who may be involved in this work.

### Limitations

The environmental scan was used to triangulate evidence from the peer-reviewed literature, domain experts, and a grey literature search. There are some limitations associated with this type of environmental scan methodology. First, the search was limited to research articles and grey literature on PPMA services across Canada only. This was done to streamline our efforts by focusing our analysis on PPMA features in the Canadian context. Studies were not excluded on the basis of their methodological quality. Second, a portion of information gathering about other PPMA programs in Canada included web searches and databases, which are only as accurate and up-to-date as the information posted on them. Third, a portion of this information was attained through personal communication with individuals who represent various provincial organizations. As such, reproducing the research process may be a challenge. Fourth, despite providing a definition of “minor ailment” at the start of the process, considerable variation was encountered across jurisdictions, ranging from differences in prescribing authority and naming of the services that describe them to diversity in the range of conditions considered to be minor ailments. For example, fungal skin infections are referred to more broadly in some jurisdictions but referred to more specifically by type (e.g., tinea pedis) in other jurisdictions. Another example is UTI. Some jurisdictions define this condition as “uncomplicated urinary tract infection” (e.g., New Brunswick, Ontario), some as “cystitis—acute, uncomplicated” (e.g., Saskatchewan), while others define it as “uncomplicated cystitis” (e.g., Manitoba, Nova Scotia). Furthermore, provinces like Nova Scotia consider prescribing for UTIs under a separate component of the legislation titled “Prescribing for a Diagnosis Supported by a Protocol” rather than under their minor and common ailment regulations [21]. The resulting ambiguity was a limitation of our process as it interfered with our ability to draw accurate comparisons between jurisdictions and apply the generalizations required for a clear summary.

## 5. Conclusions

As pharmacy practice continues to evolve to meet the current and future demands of healthcare, including public health emergencies such as COVID-19, so must the programs (e.g., PPMA services) that enable pharmacists to provide patient care. Pharmacists actively participated in the pandemic response, which highlighted their role and commitment to accessible healthcare. PPMA services offer another opportunity for pharmacists to provide streamlined care pathways for patients.

This paper explored the development of the PPMA service in Ontario from the perspectives of those involved in its design and implementation. Based on our experience, we found that taking an outcomes-focused, systems-based approach by considering the objectives of the program (i.e., improved access to care in the community) and by engaging with stakeholders throughout the healthcare system allowed us to identify key components to the program, identify areas of greatest need, and ensure that quality and safety remain at the core.

Through this work, we are able to offer an academic and policy perspective that may help other policy makers and individuals involved in effective pharmacy service design and implementation. Use of the frameworks (i.e., COM-B, Logic Model) was found to offer valuable insight as explanatory and diagnostic tools in policymaking; these frameworks present an alternative approach to PPMA program design and implementation that considers the potential barriers and enablers while visualizing the objectives and intended outcomes that can be used as a guide for evaluative studies. This also enabled the review of pharmacy curricula to ensure that the components of the PPMA program (e.g., physical and non-physical assessment) are covered in universities prior to licensure and service delivery.

Continual evaluation and review of programs may help inform enhancements to the services necessary to meet the changing demands of healthcare in the years to come. This requires the generation of additional high-quality evidence through the use of adequately powered randomized controlled trials or longitudinal observational studies evaluating PPMA programs, which are focused on the use of validated clinical outcome measures [50]. The availability of this type of evidence provides a foundation for informing changes to programs based on emerging healthcare needs. Thus, the PPMA programs should be able to adapt to the dynamic healthcare system and mounting body of evidence, for example, adding conditions to the program in light of new literature proving cost effectiveness and optimal clinical outcomes. Furthermore, additional research may provide robust evaluation data necessary for the comprehensive appreciation of the impact of existing PPMA programs both nationally and internationally.

## Figures and Tables

**Table 1:** Conditions authorized for pharmacist prescribing under minor ailment programs in Canada.

Conditions ^♾^	Alberta *	Manitoba	New Brunswick	Newfoundland and Labrador	Nova Scotia	Prince Edward Island	Quebec	Saskatchewan	Ontario **
Acne vulgaris ^a^	X	X	X	X	X	X	X	X	
Acute mountain sickness prevention	X						X		
Allergic conjunctivitis	X						X	X	X
Allergic rhinitis	X	X	X	X	X	X	X	X	X
Atopic dermatitis (eczema) ^b^	X	X	X	X	X	X	X	X	X
Bacterial conjunctivitis	X							X	X
Calluses and corns	X	X ^1^	X	X	X	X			
Contraception (hormonal)	X	X ^1^			X ^3^		X	X	
Contact allergic dermatitis ^c^	X	X	X	X	X	X	X		X
Cough	X		X	X	X	X			
Candidal stomatitis (oral thrush) ^d^	X	X	X	X	X	X	X	X	X
Dandruff	X		X	X	X	X			
Diaper rash/dermatitis, irritant and candidal	X						X	X	
Diarrhea (noninfectious)	X		X	X	X	X			
Dysmenorrhea ^e^	X	X ^1^	X	X	X	X	X	X	X
Dyspepsia (indigestion)	X		X	X	X	X	X		
Emergency contraception	X	X ^1^	X	X	X	X	X	X	
Erectile dysfunction	X							X	
Folliculitis	X							X	
Fungal infections of the skin ^f^	X		X	X	X	X	X		
Gastroesophageal reflux disease (heartburn)	X	X ^1^	X	X	X	X	X	X	X
Head lice	X						X		
Headache ^g^	X		X	X	X	X		X	
Hemorrhoids ^h^	X	X	X	X	X	X	X	X	X
Herpes labialis/simplex (cold sores)	X	X ^1^	X	X	X	X	X	X	X
Herpes zoster (shingles) prevention	X	X ^1^		X ^2^	X ^4^	X ^5^	X	X	
Impetigo	X	X ^1^	X	X	X			X	X
Influenza treatment	X						X	X	
Insect bites ^i^	X		X	X	X	X		X	X
Irritant contact dermatitis ^c^	X	X		X					X
Joint pain (minor or mild) ^j^	X		X	X	X	X			
Malaria prevention	X				X ^3^		X		
Mouth/oral/aphthous ulcers ^k^	X	X	X	X	X	X	X	X	
Muscle pain (minor or mild) ^j^	X		X	X	X	X		X	X
Nasal congestion ^p^	X		X	X	X	X			
Nausea	X		X		X	X	X		
Nausea and Vomiting	X			X			X		
Nausea/vomiting of pregnancy ^l^	X	X					X		
Obesity	X							X	
Onychomycosis	X							X	
Pinworms/threadworms	X	X ^1^	X	X	X	X	X		
Pregnancy (requiring prenatal vitamins)	X						X		
Seborrhoeic dermatitis ^m^	X	X		X					
Sleep disorders (minor or mild) ^n^	X		X	X	X	X			
Smoking cessation/nicotine dependence ^o^	X	X	X	X	X	X	X	X	X
Sore throat ^p^	X		X	X	X	X	X		
Tinea corporis infection (ringworm) ^f^	X	X ^1^	X	X	X	X	X	X	
Tinea cruris infection (jock itch) ^f^	X	X ^1^	X	X	X	X	X	X	
Tinea pedis infection (athlete’s foot) ^f^	X	X	X	X	X	X		X	
Upper respiratory tract conditions ^q^	X		X	X					
Urinary tract infection (UTIs) ^r^	X	X ^1^	X		X ^4^	X	X	X	X
Urticaria ^i^	X	X	X	X	X	X	X		X
Vaginal candidiasis (yeast infection)	X	X ^1^	X	X	X	X	X		
Vasomotor rhinitis	X	X							
Viral conjunctivitis	X								X
Warts ^s^	X	X ^1^	X	X	X	X			
Xerophthalmia (dry eyes)	X		X	X	X	X			

* Pharmacists with their APA (additional prescribing authorization) in Alberta can prescribe for conditions beyond what is represented in this table. ** Ontario conditions pending regulatory approval by the government. ^1^ Proposed prescriptive authority (not yet authorized); ^2^ Prescribing allowed under Newfoundland and Labrador Regulations Appendix B—Prescribing for a Preventable Disease; ^3^ Prescribing allowed under Nova Scotia Regulations Appendix E—Prescribing Preventative Medicines; ^4^ Prescribing allowed under Nova Scotia Regulations Appendix G—Prescribing for a Diagnosis Supported by a Protocol; ^5^ Prescribing allowed under Prince Edward Island Regulations Schedule A—diseases for which a vaccine may be prescribed and administered with special authorization; ^a^ Defined as mild acne in New Brunswick, Newfoundland and Labrador, Nova Scotia, Prince Edward Island, and Saskatchewan; mild/minor acne in Quebec; ^b^ Defined as mild to moderate eczema in Quebec, New Brunswick, Nova Scotia, and Prince Edward Island and as mild to moderate atopic dermatitis in Newfoundland and Labrador; ^c^ Defined as contact dermatitis by Manitoba and Newfoundland and Labrador; ^d^ Defined as oral thrush in Saskatchewan and Ontario; thrush in New Brunswick; oral candidiasis in Newfoundland and Labrador; oral fungal infection (thrush) in Nova Scotia and Prince Edward Island; thrush consecutive to the use of corticosteroid inhaler in Quebec; ^e^ Defined as pre-menstrual and menstrual pain in New Brunswick; ^f^ Defined as skin fungal infections in New Brunswick; fungal infection of the skin in Nova Scotia, Prince Edward Island and Newfoundland and Labrador; defined as tinea infection (corporis, cruris, pedis) in Saskatchewan; ^g^ Defined as mild headache in New Brunswick, Nova Scotia, Prince Edward Island and Newfoundland and Labrador; ^h^ Defined as unspecified hemorrhoids without complication in Manitoba; ^i^ Defined as hives, bug bites and stings in New Brunswick; mild urticaria (including bites and stings) in Nova Scotia, Prince Edward Island and Newfoundland and Labrador. In Ontario, includes tick bites; ^j^ Defined as minor joint pain and minor muscle pain in New Brunswick, Nova Scotia, Prince Edward Island and Newfoundland and Labrador; defined as musculoskeletal strains and sprains in Saskatchewan and Ontario; ^k^ Defined as mouth ulcers in Quebec, oral ulcers in New Brunswick, Nova Scotia, and Prince Edward Island; recurrent oral aphthae in Manitoba; aphthous ulcers in Newfoundland and Labrador; oral aphthous ulcer in Saskatchewan; ^l^ Defined as vomiting of pregnancy (unspecified) in Manitoba and defined as nausea and vomiting of pregnancy in Quebec; ^m^ Defined as seborrheic dermatitis (excluding pediatric) in Manitoba and seborrhea in Newfoundland and Labrador; ^n^ Defined as mild insomnia in Newfoundland & Labrador; ^o^ Defined as tobacco cessation in Saskatchewan. In some provinces (e.g., Ontario), prescribing for smoking cessation and nicotine dependence is included under separate legislation from prescribing for ambulatory conditions; ^p^ Defined as sore throat (excluding strep throat) in nova scotia and upper respiratory conditions (mild—cough, nasal congestion, sore throat) in Newfoundland and Labrador; ^q^ Defined as upper respiratory tract conditions (cough, nasal congestion and discharge, sore throat, fever, headache, malaise) in New Brunswick and upper respiratory conditions, mild (cough, nasal congestion, sore throat) in Newfoundland and Labrador; ^r^ Defined as urinary tract infection in women in Quebec; uncomplicated urinary tract infection in New Brunswick and Ontario; uncomplicated cystitis in Manitoba, Nova Scotia and Prince Edward Island; cystitis—acute, uncomplicated in Saskatchewan; ^s^ Defined as warts (excluding facial and genital) in New Brunswick, Nova Scotia, and Prince Edward Island; viral skin infections (common and flat warts) in Newfoundland and Labrador. **^♾^** Conditions for which pharmacists are authorized to prescribe Schedule I agents (drugs that require a prescription for sale pursuant to an assessment by a healthcare provider who is authorized to prescribe).

## Data Availability

Data related to this article can be found at https://www.ocpinfo.com/wp-content/uploads/2020/12/Minor-Ailments-Advisory-Group-MAAG-Summary-of-Recommendations-Pharmacist-Prescribing.pdf.
